# Malaria pervasiveness in Sub-Saharan Africa: Overcoming the scuffle

**DOI:** 10.1097/MD.0000000000040241

**Published:** 2024-12-06

**Authors:** Esther Ugo Alum, Tabussam Tufail, Peter Chinedu Agu, Dorcas Ibukun Akinloye, Israel O. Obaroh

**Affiliations:** a Department of Research and Publications, Kampala International University, Kampala, Uganda; b Department of Biochemistry, Faculty of Science, Ebonyi State University, Abakaliki, Nigeria; c School of Food and Biological Engineering, Jiangsu University, Zhenjiang, China; d University Institute of Diet and Nutritional Sciences, University of Lahore, Lahore, Pakistan; e Department of Biochemistry, College of Science, Evangel University, Akaeze, Ebonyi State, Nigeria; f Department of Biochemistry, College of Biosciences, Federal University of Agriculture, Abeokuta, Nigeria; g Department of Biological and Environmental Sciences, School of Natural and Applied Sciences, Kampala International University, Kampala, Uganda.

**Keywords:** anopheles mosquito, artemisinin, insecticide-treated bed nets, malaria, *Plasmodium falciparum*, Sub-Saharan Africa, vector control

## Abstract

Malaria has posed a momentous health and economic burden to the Sub-Saharan African region. The Sub-Saharan African region accounts for more than 90% of global malaria-related mortality and morbidity. Pregnant women and children under 5 years old are the most vulnerable. Mosquitoes transmit the plasmodium which is the parasite responsible for malaria. The climatic conditions, poverty, and poor healthcare system of the Sub-Saharan African region are some factors fueling this menace. There have been concerted efforts to annihilate malaria but the scuffle has been a tedious one. Malarial eradication campaigns have been focused on mosquito control through the use of insecticide-treated bed nets, use of indoor insecticide sprays, and use of larvicides. The use of artemisinin in combination with other drugs has been effective to some extent. Despite the aforementioned strategies, the pervasiveness of malaria infection in the Sub-Saharan African region is worrisome. Thus, strengthening the already existing control measures, finding novel measures through intensive research, and embracing malaria vaccination could help accelerate the overcoming of this scuffle. In this review, we utilized relevant published data from various databases to reexamine the factors fueling malaria pervasiveness in this region and spelled out point-by-point intervention protocols to end malaria scuffle.

## Graphical abstract

**Figure:**
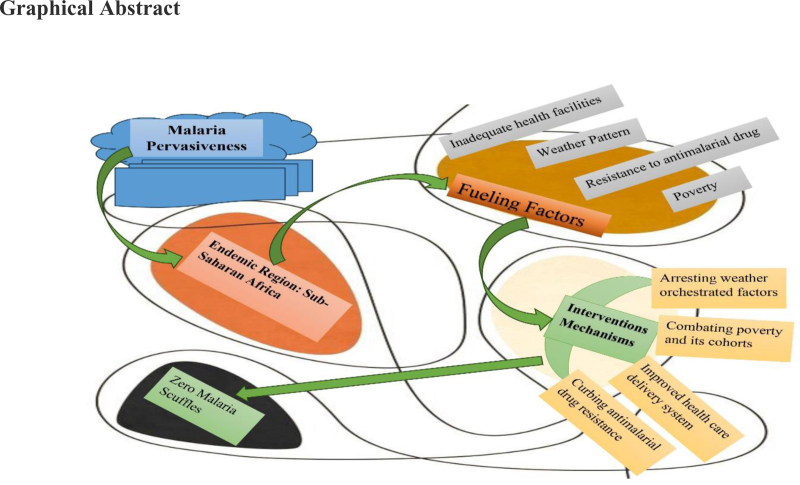

## 1. Introduction

Malaria is an ailment induced by a bite from a female anopheles mosquito. The female anopheles mosquito transfers the malarial parasite (*Plasmodium falciparum*) through a bite to the human bloodstream.^[1]^ The parasites find their way to the human liver which is its maturity domain and then back to the bloodstream where they lash to the red blood cells.^[2]^ The parasite that transmits malaria in humans is of 4 categories namely: *Plasmodium falciparum, Plasmodium vivax, Plasmodium malariae*, and *Plasmodium ovale*. However, *Plasmodium falciparum* is the most significant one, and over 99% of malaria cases in Africa are attributed to it.^[3]^ Fever and chills are the most common malaria symptoms. About 90% of all malaria-related deaths happened in Sub-Saharan Africa (SSA).^[4]^ The climate of the SSA region favors *Plasmodium falciparum* inhabitation coupled with the poor healthcare system saddling this region. In 2020, about 241 million people were infected with malaria, and 627,000 deaths caused by malarial infection occurred globally. This figure represents an increase of 14 million and 69,000 new cases and deaths, respectively, compared to 2019 data. The COVID-19 pandemic could have contributed to this upsurge as there was a disruption in malaria eradication services during the pandemic.^[5]^ Children and pregnant women have a higher risk of having severe malaria complications. It is reported that individuals who are frequently bitten by female anopheles mosquitoes possess some immunity and resistance to malarial infection. Thus, individuals from non-mosquito regions who travel to countries where the anopheles mosquito is present are more prone to severe malaria attacks.^[6]^ Further, inadequate healthcare facilities can escalate the risk and severity of malaria.^[2]^ This could be the reason for the escalated incidence of malaria in African countries.

The malarial infection has been a great burden on the healthcare and economic sectors in SSA. Malaria contributes to the high maternal and infant mortality in SSA as pregnant women and children under 5 years have lower immunity.^[7]^ The malaria pandemic in SSA has contributed to slow economic development compared to other developed countries. Households spend money out of their meager income to treat malaria making them poorer. Malaria infection is one of the reasons for absenteeism in school among children and out-of-work duties in adults. Absenteeism in school enhances out-of-school children’s numbers leading to individuals with an amputated contribution to economic growth in the future. Shortened work time due to malaria in adults leads to lower productivity.^[8]^

The high rate of malaria could also contribute to the human immunodeficiency virus (HIV) widespread in SSA. HIV infection is more common in the SSA.^[9]^ Just like malaria, women and children have a higher risk of HIV infection.^[10]^ Malaria induces anemia in infected individuals, especially children. Undergoing blood transfusion is the fastest way of managing anemia. Regrettably, blood transfusion is one of the routes of HIV transmission.^[11]^

Despite the destructiveness of malaria, it is preventable and treatable. As far back as the 1960s, chloroquine, an anti-malaria drug has been in use in Africa and it was effective until the 1970s when the malaria parasite developed resistance to chloroquine resulting in a rise in the pandemic.^[12]^ After several pieces of research, artemisinin-based combination malaria therapy emerged and has proven effective in the treatment of malaria to date.^[13]^ The use of sleeping bed nets treated with insecticides can reduce exposure to mosquitoes and thus a good malarial prevention approach.^[14]^ Recently, the malaria vaccine has been approved by the World Health Organization for children in the most vulnerable regions.^[5]^ Despite several antimalarial efforts, SSA countries are struggling to get off the hook of malaria. In this study, we shall enumerate the factors fueling malaria pervasiveness in this region and spell out point-by-point intervention protocols.

## 2. Factors fueling malaria pervasiveness in SSA

### 2.1. Weather pattern

Mosquitoes and the malarial parasite (*Plasmodium falciparum*) flourish in temperatures 77°F to 86°F (25°C–30°C) thus, the SSA countries provide a flawless abode for them.^[15]^ Mosquitoes are very sensitive to climate change as their lifecycles are affected by climate conditions. The SSA is experiencing severe climate change evidenced by the increase in the hotness of these regions. Expectedly, this rise in hotness favors mosquitoes and the malarial parasite culminating in a higher prevalence of malaria in SSA.^[16]^ Mosquitoes reproduce in stagnant water. Stagnant water is a common locus in SSA. The fervent rainfall in SSA promotes the availability of stagnant water and hence a breeding place for mosquitoes.^[17]^

### 2.2. Inadequate healthcare facilities

The standard global ratio of health workers versus people is 9.3:1000. America has 24.8:1000 health workers versus 1000 people. Regrettably, a ratio of 2.3 health workers per 1000 people is obtained in Africa.^[18]^ Inadequate health workers in SSA contribute to the high prevalence and severity of malaria in this region. Residents of SSA regions are saddled with misdiagnosis, poor diagnosis, and overdiagnosis of malaria due to a shortage of properly trained health workers. While some people take solace in the frequent use of antimalarial drugs which is tantamount to overdiagnosis, it is worthy to note that overuse of antimalarial medications can lead to an increase in antimalarial drug resistance.^[19]^ The incessant proliferation of drug vendors is not helping malaria eradication in this region. Residents patronize the drug vendors rather than seek help from trained health workers.^[20]^ Drugs purchased from these vendors sometimes are ineffective due to poor disease diagnosis and low-quality drugs yet residents still patronize them stating proximity, lower cost, and availability as their reasons.^[21]^ The study by Nshakira et al^[22]^ in Eastern Uganda reported that although 94% of children who had malaria were given chloroquine, only 34% of these children were given the standard guidelines. Children were not also given the recommended doses.

Due to the inadequate number of health workers, high cost of drugs, and long distance to hospitals, some people resort to the use of herbal medication in the treatment of malaria. The use of herbs in the maintenance of health is an age-long practice commonly utilized by rural residents.^[23,24]^ There is ample corroboration of the antimalarial properties of some plants.^[25,26]^ These plants are able to exhibit these antimalarial and other pharmacological effects by virtue of the chemical constituents ingrained in them.^[27–29]^ Despite the benefits of these herbs in the treatment of malaria, proper dosing is advocated as toxicity due to abuse is imminent.

### 2.3. Resistance to antimalarial drugs

*Plasmodium falciparum*, the parasite responsible for malaria develops resistance to antimalarial drugs after a while. This is due to the mutation of its genes to more virulent ones armed with resistance to antimalarial drugs. Chloroquine was the drug of choice in malaria treatment 5 to 6 decades ago but was replaced with sulphadoxine and pyrimethamine in the early 1970s due to resistance. There are reports of sulphadoxine and pyrimethamine inefficacy due to resistance leading to the adoption of artemisinin-based combination malaria therapy (ACT) which has minimal resistance.^[13]^ Unfortunately, ACT is more expensive than other antimalarial drugs and thus beyond the reach of some Africans who have poor economic strength. Worthy of note is the fact that the mosquitoes have also developed some resistance to those insecticides that they were susceptible to earlier. Sleeping under insecticide-treated bed nets (ITN) is one of the key measures of malaria prevention especially in areas where mosquitoes are inevitable. Resistance to antimalarial drugs plus resistance to insecticides by mosquitoes has contributed to the perpetuation of malaria prevalence in SSA.^[30]^

### 2.4. Poverty

SSA is unarguably one of the poorest in the global economic map. Poverty is one of the key antagonists in winning the battle against malaria. Some families in SSA lack the financial strength required to win this battle. This is because they cannot bear the cost of ACT, the most effective antimalarial therapy, and ITN. Consequently, malaria infection continues to ravage individuals in such classes. The use of ITN is one of the efficient prevention strategies for malaria. According to Duguma et al^[31]^ in their recent study of malaria prevalence and risk factors in Southwest Ethiopia, there was a 16.9% prevalence of malaria infection among individuals who were not using ITN compared to a 3.8% risk in users of ITN. In the same study, the prevalence of malaria among illiterates was higher compared to their learned counterparts. This corroborates the fact that education reduces the prevalence of malaria as educated individuals have better knowledge of malaria prevention and control measures. Poor education status is affiliated with poverty as education enhances income. Poor housing system orchestrated by poverty promotes malaria pervasiveness in SSA.^[32]^ Additionally, lack of money fuels delays in seeking Medicare, unguided use of herbal concoctions, and purchase of drugs from unauthorized vendors which are inimical to malaria eradication.^[33]^ Food insecurity and its accompanying undernutrition are a common threat to SSA. Its consequences are more severe in children and pregnant women. Poverty and undernutrition are 2 inseparable duos. Undernutrition is linked with depressed immunity, anemia, and severity of existing morbidities including malaria.^[34,35]^

## 3. Point-by-point intervention mechanisms

### 3.1. Tackling weather-orchestrated factors

Vector control through the use of ITN and indoor residual with insecticides plus making ACT available at a subsidized cost are the easiest ways to combat malaria menace in SSA. The exceptional strides of ITN use in the inhibition of malaria spread have by validated by several studies.^[32,36,37]^ Due to the weather pattern of the SSA, mosquito breeding is very difficult to annihilate, especially with the deleterious climate change effects in SSA which is even favoring mosquito habitats. Therefore, reducing contact with the mosquito with humans is an antidote to this quagmire. Further, the use of larvicides is a useful tool that would disrupt mosquito breeding and this is recommended by the World Health Organization as a cheaper mosquito control alternative when ITN is in short supply.^[38]^ Fillinger et al^[39]^ reported a 31% decline in malaria infection due to a one-year larvicide application in Tanzania. Cleaning up stagnant water and other mosquito breeding sites are good vector control measures that should be amplified. A study by Tsegaye et al^[37]^ in the Wogera district, Ethiopia reported that children who reside in households surrounded by stagnant water have higher odds of malarial infection. The weekly and monthly compulsory environmental sanitation exercises adopted by some neighborhoods is a virtue that should be replicated in all districts in Africa. Activities carried out during this compulsory sanitation exercise include clearing surrounding bushes, cleaning drainages, removal of stagnant water, and general fumigation. All these are aimed at controlling mosquito reproduction.

### 3.2. Combating poverty and its cohorts

There is no doubt that individuals with low financial strength are more prone to malaria attacks because they are most unlikely able to afford mosquito control tools like ITN, indoor spray insecticides, and even ACT when infected. Thus, the free distribution of free ITN, and subsidizing ACT cost will help individuals in this category. Malarial has been nick-named “poverty disease” because most of its predisposing factors are centered around poverty.^[40]^

In Southwestern Uganda, the prevalence of malaria disease decreased from 43% in 2004 to 23% in rural zones in 2010. The reason for the decline was attributed to an increase in ITN used by families.^[36]^ Malarial control and eradication campaigns should be amplified. They should be integrated into the school curriculum. This will empower residents of SSA with proper malaria control knowledge right from the cradle.

In a recent pooled prevalence and risk factors of malaria among children under 5 in 13 SSA countries, it was observed that large family size, low income of parents, low education status of mothers, and poor housing system enhanced the risk of malaria in children under 5 years.^[41]^ All these factors are poverty-orchestrated. Women’s education in SSA has been hampered by the unrefined cultural norms of confining women to home management. Unfortunately, uneducated mothers lack malarial control and treatment knowledge, and thus their children and households are at a higher risk of malaria infection.^[42]^

Depressed immunity enhances the severity of malaria in infected individuals. Poverty is associated with poor dietary intake which can build up to depressed immunity and anemia, especially in malaria’s most vulnerable group (pregnant women and infants). This can be curbed through the free distribution of nutritional supplements to poor SSA residents. More so, households should be encouraged to eat more local delicacies than refined packaged foods. Most local delicacies are endowed with more nutrients than refined packaged foods. Africa is adorned with numerous natural fruits and vegetables which it fails to harness. Fruits and vegetables are sources of nutrients with many health benefits. Some of the health benefits include blood-boosting,^[26]^ antioxidant,^[43]^ anti-inflammatory,^[44]^ anti-diabetic,^[45,46]^ immune boosting,^[47]^ and cardioprotective.^[48]^ Utilizing the surplus presence of these natural resources will no doubt curb poor nutritional status and promote immunity.

### 3.3. Enhancing healthcare delivery system

SSA leaders should brace up in the area of healthcare. The healthcare system should be adequately funded to cater to the health needs of its citizens. The ratio of health workers to citizens should be increased. This can be done by subsidizing the cost of studying health-related courses or even making it completely free for young citizens who have the mental capacity to study those courses. This will increase the number of health workers to cater to the health needs of the populace. Additionally, boosting healthcare infrastructure and the welfare needs of health workers will no doubt reduce the mass exodus of African health workers to developed countries. ACT costs should be subsidized so that households with low financial muscle can afford malaria treatment. Lowering the cost of antimalarial drugs and making them readily available will curb the incessant patronage of unauthorized drug vendors and the unguided use of herbal concoctions.

### 3.4. Curbing antimalarial drug resistance

Antimalarial drug resistance has been a drawback in the malarial eradication strides. Chloroquine is the first antimalarial drug but its use has become obsolete due to the chloroquine-resistance to malaria. Sulphadoxine and pyrimethamine replaced chloroquine until the 1970s when the malarial parasite became resistant to it. The emergence of ACT gave a huge relief to the malaria pandemic. ACT is simply the combination of artemisinin with other drugs in malaria treatment. Regrettably, artemisinin resistance has been widely reported thereby decelerating the pace of malarial eradication strides.^[49]^ Therefore, there is a dire need for new antimalarial drugs and researchers have been wide awake. There is synergy between artemisinin and other combined drugs in ACT treatment protocol. So, each drug targets different metabolic sites of the parasite. The combined action of the combined drugs reduces the incidence of resistance. Recognition of specific drug-resistant genetic molecular markers will help understand antimalarial drug resistance and predict the areas for further probe. Interestingly, scientists have identified some genetic molecular markers of drug resistance for some antimalarial drugs.^[50]^ Thus, continuous research for the identification of more markers of antimalarial drug resistance will help in formulating more efficacious antimalarial drugs. More so, the identification of new antimalarial target sites can help in curbing the high incidence of resistance. Taken together, the need for boosting malarial research cannot be over-emphasized as this research requires massive capital.

## 4. Conclusion

The fight against malaria has been a challenging one marked with progress and setbacks. Prevention of malaria through the use of malarial parasite control mechanisms like insecticide-permeated bed nets, spraying of insecticides indoors, cleaning up stagnant water, and the use of larvicides to minimize mosquito breeding has been an ancient practice with good results. The current use of ACT and vaccination has been useful. The climatic condition of SSA, low economic stand, grossly inadequate healthcare system, and the recent resistance of malarial parasite to ACT, have been antagonists to the expected malarial annihilation progress. Therefore, strengthening already existing malarial parasite control mechanisms, developing new and more effective drugs and vector control strategies by funding malarial research, embracing the malaria vaccine, improving healthcare facilities, and amplifying malarial eradication education campaigns, can help minimize the health and economic burden imposed on SSA by malaria.

## Acknowledgments

We are grateful to Kampala International University for its support.

## Author contributions

**Conceptualization:** Esther Ugo Alum.

**Validation:** Esther Ugo Alum.

**Visualization:** Esther Ugo Alum, Peter Chinedu Agu, Dorcas Ibukun Akinloye.

**Writing – original draft:** Esther Ugo Alum, Tabussam Tufail, Peter Chinedu Agu, Dorcas Ibukun Akinloye, Israel O. Obaroh.

**Writing – review & editing:** Esther Ugo Alum, Tabussam Tufail, Peter Chinedu Agu, Dorcas Ibukun Akinloye, Israel O. Obaroh.

**Data curation:** Tabussam Tufail.

**Methodology:** Tabussam Tufail, Dorcas Ibukun Akinloye.

**Resources:** Peter Chinedu Agu, Dorcas Ibukun Akinloye, Israel O. Obaroh.

**Supervision:** Israel O. Obaroh.
